# [Retracted] Downregulation of thymopoietin by miR‑139‑5p suppresses cell proliferation and induces cell cycle arrest/apoptosis in pancreatic ductal adenocarcinoma

**DOI:** 10.3892/ol.2024.14623

**Published:** 2024-08-12

**Authors:** Huadong Zhou, Linfei Zhang, Huahua Tu

Oncol Lett 18: 3443–3452, 2019; DOI: 10.3892/ol.2019.10679

Following the publication of this paper, it was drawn to the Editor's attention by a concerned reader that certain of the colony formation assay data shown in Fig. 4C on p. 3448 had already appeared in an article written by a different author that had already been accepted for publication [Ma Q: MiR-219-5p suppresses cell proliferation and cell cycle progression in esophageal squamous cell carcinoma by targeting CCNA2. Cell Mol Biol Lett 5: 24, 2019.]

Owing to the fact that the contentious data in the above article had already been accepted for publication prior to its submission to *Oncology Letters*, the Editor has decided that this paper should be retracted from the Journal. The authors were asked for an explanation to account for these concerns, but the Editorial Office did not receive a reply. The Editor apologizes to the readership for any inconvenience caused.

